# Implications of High-Density Cholesterol Metabolism for Oocyte Biology and Female Fertility

**DOI:** 10.3389/fcell.2022.941539

**Published:** 2022-09-14

**Authors:** Andreina Arias, Alonso Quiroz, Nicolás Santander, Eugenia Morselli, Dolores Busso

**Affiliations:** ^1^ Laboratory of Nutrition, Metabolism and Reproduction, Research and Innovation Center, Program of Reproductive Biology, Universidad de Los Andes, Santiago, Chile; ^2^ Faculty of Biological Sciences, Pontificia Universidad Católica de Chile, Santiago, Chile; ^3^ School of Medicine, Pontificia Universidad Católica de Chile, Santiago, Chile; ^4^ Instituto de Ciencias de la Salud, Universidad de O’Higgins, Rancagua, Chile; ^5^ Department of Basic Sciences, Faculty of Medicine and Sciences, Universidad San Sebastián, Santiago, Chile; ^6^ IMPACT, Center of Interventional Medicine for Precision and Advanced Cellular Therapy, Santiago, Chile

**Keywords:** female fertility, high-density lipoprotein metabolism, cholesterol, unesterified cholesterol, oocyte

## Abstract

Cholesterol is an essential component of animal cells. Different regulatory mechanisms converge to maintain adequate levels of this lipid because both its deficiency and excess are unfavorable. Low cell cholesterol content promotes its synthesis and uptake from circulating lipoproteins. In contrast, its excess induces the efflux to high-density lipoproteins (HDL) and their transport to the liver for excretion, a process known as reverse cholesterol transport. Different studies suggest that an abnormal HDL metabolism hinders female fertility. HDL are the only lipoproteins detected in substantial amounts in follicular fluid (FF), and their size and composition correlate with embryo quality. Oocytes obtain cholesterol from cumulus cells *via* gap junctions because they cannot synthesize cholesterol *de novo* and lack HDL receptors. Recent evidence has supported the possibility that FF HDL play a major role in taking up excess unesterified cholesterol (UC) from the oocyte. Indeed, genetically modified mouse models with disruptions in reverse cholesterol transport, some of which show excessive circulating UC levels, exhibit female infertility. Cholesterol accumulation can affect the egg´s viability, as reported in other cell types, and activate the plasma membrane structure and activity of membrane proteins. Indeed, in mice deficient for the HDL receptor Scavenger Class B Type I (SR-B1), excess circulating HDL cholesterol and UC accumulation in oocytes impairs meiosis arrest and hinders the developmental capacity of the egg. In other cells, the addition of cholesterol activates calcium channels and dysregulates cell death/survival signaling pathways, suggesting that these mechanisms may link altered HDL cholesterol metabolism and infertility. Although cholesterol, and lipids in general, are usually not evaluated in infertile patients, one study reported high circulating UC levels in women showing longer time to pregnancy as an outcome of fertility. Based on the evidence described above, we propose the existence of a well-regulated and largely unexplored system of cholesterol homeostasis controlling traffic between FF HDL and oocytes, with significant implications for female fertility.

## Introduction

### Overview of Cholesterol Biochemistry and Whole-Body Metabolism

Cholesterol, the principal sterol in animals, comprises a four-ring structure and a single hydroxyl group attached to one of the rings (Cortes et al., 2013). This essential component of cell membranes and precursor of steroid hormones, bile acids, and vitamins, among other molecules, exists in two forms: free or unesterified cholesterol (UC) and cholesteryl ester (CE) or esterified cholesterol (Luo et al., 2020). The first is an amphipathic, active form present in cell membranes. The second inactive form results from esterification of UC with fatty acids for storage in lipid droplets (LD) or lipoproteins.

Cholesterol is synthesized by almost every cell type and provided by the diet. The liver delivers both endogenously synthesized and exogenously acquired cholesterol to the bloodstream for its distribution to other tissues by secreting lipoproteins. These specialized particles solubilize lipids in blood and allow their bidirectional exchange with cells (Salter and Brindley, 1988). Lipoproteins are classified, according to their density, into chylomicrons, very-low-density lipoproteins (VLDL), low-density lipoproteins (LDL), and high-density lipoproteins (HDL). Each lipoprotein class contains different proportions of lipids and proteins (apolipoproteins) that determine their structure, specificity towards specific receptors and functional properties (Salter and Brindley, 1988). Apolipoprotein B (Apo B) is the principal protein component of LDL and VLDL. This large and hydrophobic protein assembles with lipids in the endoplasmic reticulum (ER) of liver or intestinal cells (Jonas et al., 2008). Smaller apolipoproteins (e.g., Apo A1, Apo A2, Apo E) have much smaller molecular masses, they can solubilize in blood, acquire lipids, and modulate the activity of plasma enzymes (Jonas et al., 2008). Although lipoproteins have mainly been studied in plasma, these particles are also present in other body fluids such as cerebrospinal, amniotic, and follicular fluid (FF), etc (Blackett and McConathy, 1982; Suchanek et al., 1988; Mahley, 2016).

In different tissues, a low cholesterol level promotes its own synthesis and uptake from circulating lipoproteins, whereas its excess induces efflux to HDL and excretion through the liver, the key organ regulating cholesterol metabolism (Luo et al., 2020). The mechanism of cholesterol mobilization from extrahepatic tissues to the liver for excretion is denominated reverse cholesterol transport (Luo et al., 2020). This process starts with the efflux of UC and phospholipids from the plasma membrane (PM) to Apo AI *via* ATP-binding cassette transporters A1 (ABCA1) (Brooks-Wilson et al., 1999). These lipid-poor nascent HDL particles travel in the bloodstream to other tissues and serve as acceptors for UC and other lipid classes (Phillips, 2014). Concomitantly, UC at the HDL surface is esterified by the enzyme Lecithin: Cholesterol Acyl Transferase (LCAT) using fatty acids from phospholipids and stored as CE in the lipoprotein core, along with other neutral lipids like triglycerides and hydrophobic vitamins (Frohlich et al., 1982). Mature, lipid-rich HDL mediate the efflux of additional UC cholesterol from extrahepatic cells *via* ABCG1 and finally bind to the hepatic non-endocytic Scavenger Receptor class B type 1 (SR-B1), which takes up CE for catabolism into bile acids or direct biliary excretion (Acton et al., 1996).

In addition to the liver, SR-B1 is also expressed in cells that require high amounts of cholesterol for their function, such as steroidogenic cells and macrophages. In macrophages, SR-B1 provides the cell with cholesterol to regulate phagocytic functions, which is essential for infection and tissue homeostasis responses. In steroidogenic cells of the adrenal gland, the ovaries and the testis, SR-B1 takes up CE from circulating HDL, which is then transported to mitochondria. Mitochondrial enzymes of the P450 family catalyze the conversion of cholesterol into steroid hormones (e.g., corticoids in the adrenal gland, estrogen and progesterone in the ovary, and testosterone in the testis).

### Cholesterol Cellular Distribution and Homeostatic Processes

UC is distributed unevenly among membranes from different cellular organelles (Ikonen and Zhou, 2021). This distribution seems to depend on the relative affinity of cholesterol for the lipids composing each membrane. Cholesterol co-localizes preferentially with phosphatidylcholine, phosphatidylethanolamine, phosphatidylserine, and sphingomyelin, the major constituents of the PM. Thus, the proportion of UC to total lipids is high (near 40% of total lipids) in the PM and very low (less than 5%) in the ER and mitochondria. Due to the unique biophysical properties of cholesterol, its level determines the fluidity and heterogeneity of components at the PM (Ikonen and Zhou, 2021). Indeed, cholesterol-rich specialized microdomains, including caveolae, tetraspanin-enriched microdomains and lipid rafts, are crucial for regulating signaling pathways by restricting the localization and activity of specific proteins in the cell membrane.

Different convergent regulatory mechanisms maintain cell cholesterol levels within appropriate ranges because excess UC (levels above the physiological set point) is cytotoxic (Luo et al., 2020). Cholesterol accumulation in the PM induces the redistribution of this lipid to intracellular membranes and activates homeostatic responses to reduce cholesterol synthesis and uptake (Lange et al., 2014). Sterol regulatory element-binding proteins (SREBPs) are key players controlling cholesterol synthesis in the ER (Cortes et al., 2013). The active domains of these membrane-bound transcription factors are released proteolytically to enter the nucleus and activate genes promoting cholesterol synthesis and uptake. SCAP (SREBP cleavage-activating protein) is a sterol-regulated escort protein that transports SREBPs from their site of synthesis in the ER to their site of cleavage in the Golgi. When cholesterol levels are low, SREBPs upregulate cholesterol biosynthesis and uptake by increasing the transcription of the rate-limiting enzyme 3-Hydroxy-3-Methylglutaryl-CoA Reductase (HMGCR) and the PM LDL receptor (LDLR), respectively. On the other hand, when cholesterol levels are high, the cells upregulate the transcription of ABCA1 and ABCG1 and induce the degradation of cholesterol receptors at the PM (Acton et al., 1996).

Another mechanism to prevent damage by UC is the esterification of this lipid by sterol O-acyltransferase, also known as acyl-CoA cholesterol acyltransferase (ACAT), residing in the ER and storage of inactive CE within the hydrophobic nucleus of LD (Chang et al., 1997). In these organelles, fatty acids are also stored in triglycerides, an important source of ATP production *via* β-oxidation (Geltinger et al., 2020).

### Cell Toxicity by UC Excess: Proposed Mechanisms

Although the precise mechanism of UC cytotoxicity is unclear, this lipid can disturb the fluidity of membranes, disrupt lipid rafts, form cytoplasmic crystals and activate different signaling pathways, all of which can reduce the cell´s viability (Tabas, 1997).

An abnormal proportion of cholesterol at the outer leaflet of the PM can reduce its fluidity and may block the function of signaling proteins that reside in lipid rafts. In artificial monolayers composed of a mixture of cholesterol, phosphatidylcholine and sphingomyelin, a rise in the percentage of cholesterol (over 33%) impedes the formation of membrane subdomains (Milhiet et al., 2002). In the adipocyte cell line 3T3-L1, increased cholesterol in the PM dysregulates the insulin-dependent glucose transport system by reducing the amount of 4,5-bisphosphate (PIP(2))-regulated filamentous actin (F-actin) structures, finally impairing Glut-4 functionality and causing insulin resistance (Bhonagiri et al., 2011).

Consistently, membrane cholesterol concentration changes in mature neurons affect their susceptibility to cell death. In young hippocampal rat neurons, increased amounts of cholesterol in the cell membrane promote beta-amyloid-dependent calpain activation, leading to the generation of the cytotoxic tau fragment (Nicholson and Ferreira, 2009). Conversely, in mature neurons, when the cholesterol concentration in the membrane is pharmacologically reduced, calpain activation and therefore tau production is blunted, promoting cell survival (Nicholson and Ferreira, 2009). Notably, cholesterol accumulation in membranes different from the PM can also affect cell viability. In liver and neurodegenerative diseases, the chronic accumulation of cholesterol in mitochondrial membranes modulates their permeability and the release of intermembrane proteins such as Bax that activate death-inducing caspases (Montero et al., 2010). This effect of cholesterol on mitochondrial membrane permeability seems to involve a reduction of GSH transport into the mitochondrial matrix; this reduced GSH availability results in the increased generation of ROS by cell death stimuli such as TNF, hypoxia or Aβ (Montero et al., 2010).

Another mechanism of UC-mediated cytotoxicity is cholesterol crystallization in LD and PM, identified in different cell types, from phagocytes to hepatocytes (Marí et al., 2006; Duewell et al., 2010; Shu et al., 2018). During UC transport for storage in LD, the concentration of this lipid may exceed the ability of phospholipids in the LD membrane to solubilize it and cause the precipitation of cholesterol monohydrate crystals (Marí et al., 2006). Cholesterol crystals induce an inflammatory response, both in mice and humans, which is associated with the activation of the NLRP3 inflammasome and may lead to lysosomal damage, finally inducing necrosis and programmed cell-death pathways such as pyroptosis, a process characterized by inflammasome activation and secretion of inflammatory cytokines (Duewell et al., 2010; Rajamäki et al., 2010; Wree et al., 2014; Favor et al., 2021). In bone-marrow-derived macrophages, the binding of cholesterol crystals to the cell surface extracts cholesterol from the PM and promotes an inflammatory response that culminates in PM damage and necrotic cell death (Shu et al., 2018). Interestingly, macrophages exposed to increasing concentration of UC also show intrinsic and extrinsic induction of apoptosis, with a fraction of the cells showing biochemical, morphological and molecular indicators of apoptosis activation, including DNA fragmentation, externalization of phosphatidylserine at the PM, activation of pro-apoptotic pathways, i.e., Fas/FasLigand, Bax-dependent cytochrome C release from the mitochondria and subsequent caspase 9 activation (Yao and Tabas, 2000; Yao and Tabas, 2001). Thus, cellular UC overload does not affect a single cell death pathway but instead activates different mechanisms that converge to cell death. Interestingly, apoptosis in macrophages can be diminished by the depletion of SR-B1 (Galle-Treger et al., 2020).

Finally, UC accumulation can promote cell toxicity by forming oxysterols, some of which promote cell death in diverse cell types from different species (Vejux et al., 2020). In rats fed diets enriched in fat and cholesterol, oxysterols promote liver toxicity by impairing mitochondrial function and driving apoptosis in hepatocytes, a mechanism potentially involved in the progression from non-alcoholic fatty liver disease to hepatic steatosis (Bellanti et al., 2018). Interestingly, 25-hydroxycholesterol and 7-Ketocholesterol activate oxiapoptophagy, a type of cell death associated with oxysterol-mediated induction of oxidative stress and characterized by features of both apoptosis and autophagy, that has been associated with the development of different diseases ranging from atherosclerosis to neurodegeneration (Anderson et al., 2020; You et al., 2021).

### Lipoprotein Cholesterol as a Substrate for Ovarian Steroidogenesis

The implications of cholesterol for female reproduction have been suggested by different studies in various species (Skoblina et al., 1981; Shim et al., 2002; Bełtowski and Semczuk, 2010; Fujimoto et al., 2010; van Montfoort et al., 2014). Cholesterol is indispensable as a substrate for steroid hormone synthesis in the mammalian ovarian follicle. This structure is organized into two compartments, separated by a basal lamina: the outer compartment comprising the vascularized thecal cells and the inner, nonvascular compartment containing granulosa cells, the oocyte and cumulus cells, and the antrum filled with FF (Fahiminiya and Gérard, 2010). During steroidogenesis, follicular cells use cholesterol from different sources, i.e., local synthesis and uptake from lipoproteins in FF, and receptors for different lipoprotein classes are expressed in a cyclic and well-concerted manner in specific follicular cells to maximize steroid hormone production. FF is the product of granulosa secretions and small proteins and particles that filter from the blood through the basal lamina (Shalgi et al., 1973). It provides a rich microenvironment, composed of hormones, signaling factors, metabolites and nutrients, that supports oocyte development during follicular development (Revelli et al., 2009).

HDL is the major lipoprotein present in FF from different species, including bovine (Brantmeier et al., 1987), porcine (Chang et al., 1976), equine (Le Goff, 1994) and humans (Simpson et al., 1980); larger lipoproteins are barely detectable in antral follicles from those species. Existing evidence suggests that FF HDL mainly originate from plasma (Le Goff, 1994; Jaspard et al., 1997; Quiroz et al., 2020). The first evidence to support this came from early work from Le Goff and collaborators who characterized FF HDL in mares (Le Goff, 1994). In their study, plasma and FF lipoproteins were isolated by density gradient ultracentrifugation and characterized according to size, density, lipid content and electrophoretic mobility. The authors concluded that FF contained smaller HDL subtypes, with higher ratios of cholesterol/phospholipid and CE/UC than plasma. They hypothesized that larger plasma HDL particles are excluded by the basal lamina pores and smaller HDL permeate into the antrum and are metabolically transformed in the follicle to enhance reverse cholesterol transport. Consistently to what was described in equine, human FF were reported to exhibit a different composition compared to circulating HDL (Jaspard et al., 1997). Compared to plasma HDL, human FF HDL seem to be predominantly smaller, with less UC (and a higher esterification rate), more phospholipids, and a higher ApoA-IV/ApoA-I ratio, supporting the idea that HDL are either of different origin or subject to remodeling in the follicular antrum. We recently provided the first experimental evidence demonstrating that circulating HDL reach the antrum (Quiroz et al., 2020). In an ovarian cross-transplantation experiment in mice, ApoA-I was immunodetected on the surface of granulosa cells facing the antrum and within the antral cavity of ApoA-I deficient (ApoA-I KO) ovaries transplanted into wild type (WT) mice. By contrast, ApoA-I was not detected in the antrum or granulosa cells of WT mouse ovaries transplanted into Apo-AI KO females. Together, the results of our study suggested that mouse FF HDL originate from plasma. Unfortunately, we were unable to perform biochemical analyses to describe the properties of mouse FF HDL due to sampling limitations derived from the minimal volume of FF recovered in these animals. Intriguingly, ApoA-I mRNA and protein were detected in granulosa cells from chicken and human ovaries, opening the possibility that at least some FF HDL may be of intraovarian origin (Hermann et al., 1998; Choi et al., 2010).

FF HDLs, rich in esterified cholesterol, are poor substrates for steroid synthesis (Parinaud et al., 1987). Thus, during the follicular stage, thecal cells incorporate cholesterol from blood HDL and synthesize androgens (androstenedione and testosterone) which cross the basal membrane and fuel estrogen production by granulosa cells in the developing follicle. After the LH surge, the follicular basal membrane becomes more permeable to larger lipoproteins which enhance cholesterol availability to support the active production of steroids during ovulation (Simpson et al., 1980; Cherian-Shaw et al., 2009). Indeed, FF cholesterol concentrations are positively correlated with follicular size, and the proportions of LDL and VLDL in preovulatory follicles increase gradually (Argov et al., 2004). The LH rise also activates the SREBP/SCAP system and induces *de novo* cholesterol biosynthesis in mouse granulosa cells (Nakanishi et al., 2021). After ovulation, the follicle becomes vascularized and follicular cells mobilize stored CE and upregulate the expression of lipoprotein receptors (e.g., VLDLR, LDLR, SR-B1 and LRP8) (Argov et al., 2004; Cherian-Shaw et al., 2009). In the corpus luteum, luteal cells use cholesterol obtained by the novo biosynthesis and from plasma LDL and HDL to rapidly increase progesterone synthesis. In most species, SR-B1 is mainly expressed in theca and corpora lutea cells to provide cholesterol as the starting substrate for steroidogenesis (Miranda-Jiménez and Murphy, 2007; Cherian-Shaw et al., 2009; Lai et al., 2013; Chang et al., 2017). The LDLR expression in granulosa cells is mostly extinguished as luteinization progresses (Cherian-Shaw et al., 2009). By contrast, luteinization causes upregulation of SR-BI expression and posttranslational modifications that favor its insertion in luteal cell membranes (Miranda-Jiménez and Murphy, 2007; Cherian-Shaw et al., 2009). Experiments using primary cultures of rat ovarian granulosa showed that in luteal cells SR-B1 is less stringently regulated than LDLR. Hence, its increasing expression as luteinization progresses helps these cells evade the cholesterol homeostatic mechanisms and ensure sufficient cholesterol acquisition for steroid hormone production (Lai et al., 2013).

Studies by the group of A Rodriguez suggest that SR-B1 is relevant for steroidogenesis in human granulosa cells during the periovulatory stage (Velasco et al., 2006). SR-B1 expression in granulosa cells isolated from mature follicles obtained during oocyte aspirations *in vitro* fertilization (IVF) procedures shows positive associations with plasma estrogen levels, the number of oocytes retrieved, and fertilization rates (Velasco et al., 2006). Further support for this role came from manipulations of human granulosa cells *in vitro* by the same group. Indeed, reduction of SR-B1 levels using siRNA led to impaired progesterone secretion in these cells, even after stimulation with forskolin (Kolmakova et al., 2010). In addition, single nucleotide polymorphisms (SNP) in the *SCARB1* gene, which codes for SR-B1 in humans, are associated with progesterone levels in FF and viable pregnancy outcomes in women subjected to IVF (Yates et al., 2011). Altogether, the evidence indicates that lipoprotein-mediated cholesterol uptake is relevant for steroidogenesis in theca, granulosa, and luteal cells.

As mentioned above, LDL and larger lipoproteins are not abundant in FF. LDL are prone to forming oxidized LDL (oxLDL), which induce multiple metabolic and functional disturbances in cells, as shown by numerous studies in different pathologies (Mineo, 2020; Deng et al., 2022). It may be hypothesized that exposure of oocytes in preovulatory follicles to high LDL levels is avoided as a protective mechanism to prevent their damage by oxidative stress. However, apoB-containing lipoproteins are secreted by human granulosa cells and show a positive relationship with improved fertility parameters after *in vitro* fertilization (IVF) (Gautier et al., 2010). These results suggest that large lipoproteins may be locally assembled and secreted into FF. In the last years, numerous studies have agreed in demonstrating that fertility is compromised in women with high body mass index, and that one of the drivers of this dysfunction is bad oocyte quality (Silvestris et al., 2018). Women with obesity are exposed to systemic chronic oxidative stress and exhibit high levels of circulating oxLDL (Korac et al., 2021). In this regard, exposure of preovulatory follicles to high levels of oxLDL in women with obesity and the resulting adverse effects on the oocyte viability and function may be one of the mechanisms contributing to infertility in obesity. Additional studies from different perspectives are required to underscore the complex mechanisms through which an adverse cholesterol metabolism may hinder fertility in women with obesity (Gonzalez et al., 2022).

### Relationship Between FF HDL and Oocyte Quality and Developmental Potential

Early knowledge on the role of HDL particles for oocyte quality and developmental potential was provided by Fujimoto, Browne and collaborators, who analyzed FF in assisted reproductive technology (ART) procedures (Fujimoto et al., 2010). In those studies, FF HDL cholesterol concentration and particle size were negatively correlated with IVF outcomes and preimplantation embryo quality parameters, including fragmentation and symmetry (Browne et al., 2008; Browne et al., 2009; Kim et al., 2017a). The authors proposed that alterations in oocyte intracellular cholesterol homeostasis due to abnormal HDL may hinder embryo quality (Fujimoto et al., 2010). It is interesting to note that those studies only provided information on total cholesterol, so associations between UC or esterified cholesterol and the parameters mentioned above cannot be inferred from these studies. Similar to plasma HDL, FF HDL contain other molecules that may be important for the physiology of oocytes and early embryos, such as sphingosine 1-phosphate (S1P), carotenoids, vitamins and antioxidant enzymes (Schweigert, 2003; Kim et al., 2017b). Indeed, analyses of FF HDL obtained during ovum pickup preceding ART procedures show that specific molecular components from HDL of different sizes relate to preimplantation embryo quality (Schweigert, 2003; Browne et al., 2009; Kim et al., 2017b). In this context, HDL-associated molecules that may be considered potential predictors of successful IVF include γ-tocopherol, which correlates with less embryo fragmentation (Browne et al., 2009); FF-paraoxonase-arylesterase activity, a positive predictor of embryo cell number (Kim et al., 2017b); phosphatidylcholine, positively correlated with embryo cleavage (Wallace et al., 2012); and S1P, associated with good embryo quality and a better chance of clinical pregnancy (Garrido et al., 2000). In recent work, FF HDL from modified natural IVF cycles showed a positive relationship between FF HDL anti-inflammatory properties and the developmental potential of the oocyte (Jia et al., 2020). However, the relationship with pregnancy success was not significant in that study. There are some limitations in studies using samples from ART procedures, e.g., inclusion of a low number of patients with variable clinical characteristics, determination of a reduced group of molecules in each study and transfer of more than one embryo, all of which difficult direct embryo-pregnancy relationships. However, they provide valuable evidence that supports the idea that the close interaction of FF HDL and the developing oocyte is essential for its developmental potential through endocrine and non-endocrine mechanisms. Different functions attributed to FF HDL, including reverse cholesterol transport, antioxidant capacity and signaling in follicular cells and the oocyte, and provision of cholesterol as a substrate for steroid synthesis during the periovulatory phase, may be necessary for the physiology of oocytes and early embryos. The following sections will discuss how FF HDL may contribute to oocyte cholesterol homeostasis.

### Cholesterol Content and Distribution in Oocytes and Eggs

Mouse oocytes and preimplantation embryos are deficient in cholesterol biosynthesis, as shown by the low expression of transcripts encoding enzymes of the cholesterol synthetic pathway and the levels of radio-labeled cholesterol detected after providing denuded oocytes with labeled acetate (Su et al., 2008). Oocytes are most probably unable to take up cholesterol from the surrounding extracellular microenvironment because they don´t express lipoprotein receptors. Indeed, neither HDL cholesterol receptor (SR-B1) nor LDLR are expressed in mammalian oocytes (Sato et al., 2003; Trigatti et al., 2003; Quiroz et al., 2020). Since they cannot synthesize or take up cholesterol by themselves, oocytes rely on the provision of cholesterol from granulosa or cumulus cells. Data suggest that cholesterol is transported from cumulus cells to oocytes across the zona pellucida *via* gap junctions, intercellular channels that allow passage of small molecules between cells (Anderson and Albertini, 1976). These structures provide the physical basis for cumulus-to-oocyte cholesterol transfer within the follicle. Interestingly, oocytes stimulate cholesterol biosynthesis in cumulus cells through paracrine growth factors such as bone morphogenetic protein 15 (BMP15) and growth differentiation factor 9 (GDF9) (Su et al., 2008).

The cholesterol content in the oocyte seems to modulate maturation, fertilization, activation and embryo development (Skoblina et al., 1981; Shim et al., 2002; Buschiazzo et al., 2008; Buschiazzo et al., 2013; Marco-Jimenez et al., 2014; Yesilaltay et al., 2014). Buschiazzo et al. showed that cholesterol depletion with methyl-beta-cyclodextrin (MβCD) in the mouse oocyte PM reduced *in vitro* fertilization and egg activation rates, which was associated with alterations in the structure of PM microdomains (Buschiazzo et al., 2013). MβCD-treated oocytes also showed raft-associated -but not non-raft-associated- protein depletion. Interestingly, cholesterol repletion using cholesterol-loaded MβCD (MβCD-Chol) almost completely restored fertility (Buschiazzo et al., 2013). Recent studies in farm animals showed that a significant proportion of cholesterol is removed from the PM during vitrification, a well-known cryopreservation method used to preserve female gametes (Buschiazzo et al., 2017; Atalla et al., 2018; Chen et al., 2019). This method, applied in farm animal production, human-assisted reproduction and protection of endangered species, is still considered inefficient because it affects the oocytes’ viability and fertilization capacity. Studies using bovine, ovine and porcine eggs demonstrated that rising UC cholesterol levels transiently during vitrification preserves oocyte lipid rafts organization and quality, resulting in improved maturation, viability, fertilization, and early development rates after thawing (Buschiazzo et al., 2017; Atalla et al., 2018; Chen et al., 2019). In a recent paper, Hao et al. showed that the level of JUNO, a glycosylphosphatidylinositol (GPI)-anchored protein expressed on the egg surface that is essential for female fertility (Bianchi et al., 2014), was significantly reduced in bovine eggs by vitrification (Hao et al., 2021). The successive treatment of MβCD-Chol/MβCD before/after cryopreservation preserved oocyte JUNO levels and significantly improved fertilization capacity. The GPI anchor allows proteins to anchor to lipid rafts (Seong et al., 2013). Altogether, this information suggests that changes in cholesterol levels at the egg PM may affect the activity of membrane proteins, especially those localized in rafts.

In a recent study cholesterol depletion from the PM of Xenopus oocytes induced the activation of the heterologously expressed rat transient receptor potential vanilloid 4 (TRPV4) (Lakk et al., 2021). TRPV4 is a mechanosensitive nonselective cation channel, not localized in PM microdomains, that transduces mechanical stimuli, e.g., membrane strain, shear flow, swelling, and thermal stimuli, into calcium (Ca^2+^) signals that control a wide range of downstream signaling pathways (Rosenbaum et al., 2020). Ca^2+^ homeostasis is particularly relevant in mammalian eggs because intracellular Ca^2+^ oscillations trigger activation and embryo development after fertilization. Cytoplasmic Ca^2+^ levels are strictly regulated before sperm-egg fusion to prevent egg parthenogenetic activation, fragmentation and apoptosis (Gordo et al., 2002; Ozil et al., 2005). Multiple ion transporters regulate intracellular Ca^2+^ levels (iCa^2+^) and storage of Ca^2+^ in the ER, as reviewed in (Wakai et al., 2013). We showed that loading WT mouse eggs with MβCD-Chol induced an elevation of iCa^2+^ and a reduction in maturation promoting factor and MAPK activities, followed by progression to meiotic stages beyond MII, extrusion of a second polar body, and parthenogenic cleavage (Yesilaltay et al., 2014). Recent work from R. Fissore´s laboratory demonstrated that at least three divalent-permeable ion channels expressed in the egg PM regulate Ca^2+^ influx during fertilization-induced oscillations: the transient receptor potential vanilloid member 3 (TRPV3), the transient receptor potential cation channel subfamily M, member 7 (TRPM7) and the T-type channel CaV3.2 (Bernhardt et al., 2018; Mehregan et al., 2021). It may be hypothesized that cholesterol in the egg PM. Activates Ca^2+^ channels and therefore its levels need to be low to maintain MII arrest. Although this hypothesis needs to be demonstrated experimentally, several observations support this idea: 1) an increase in the proportion of cholesterol in eggs activates PM Ca^2+^ channels; 2) the expression of SOATs increases during maturation (progression from GV to MII stages); and 3) cholesterol levels decrease in mouse oocytes during this period (Li et al., 2020).

The mechanisms regulating cholesterol homeostasis by esterification and storage in oocyte LD are largely unknown. In bovine eggs, removing PM cholesterol with MβCD significantly reduced the density of LD in the cell cortex (Buschiazzo et al., 2017). On the other hand, pulse-chase experiments using fluorescent BODIPY-labelled cholesterol demonstrated the internalization of the probe and its accumulation in cytoplasmic structures resembling LD (Buschiazzo et al., 2017). This evidence suggests that hydrolysis of CE and esterification of UC may be used by oocytes to control cholesterol content in membranes, as observed in other cell types (Ouimet and Marcel, 2012). However, more studies are needed to understand how LD could help to modulate cholesterol levels in oocytes. Species-specific mechanisms need to be considered because lipid levels and LD distribution are highly variable in different animals (Bradley and Swann, 2019). Compared to human or mouse oocytes, the oocytes from dogs, pigs, cows and sheep contain a much higher lipid concentration and LD that are detectable by transmission microscopy (Bradley and Swann, 2019).

### Link Between Infertility and UC: Lessons From Genetically Modified Mice

Evidence from genetically modified mice suggests that disruptions in the HDL metabolism may affect female fertility (Table 1). One of the best described mouse models linking disruptions in HDL metabolism and infertility is the SR-B1 knock out (SR-B1 KO) mouse, generated in M. Krieger´s laboratory 25 years ago to understand the relationship between reverse cholesterol transport and atherosclerosis risk (Rigotti et al., 1997). SR-B1 KO mice display distinctively large HDL enriched in apolipoprotein E (ApoE) and UC and an increased incidence of pathologies in critical physiological systems, including high atherosclerosis susceptibility (Rigotti et al., 1997; Rigotti et al., 2003; Hoekstra et al., 2010; Liu et al., 2021). Unexpectedly, during the generation of SR-B1 KO mice females were found to be sterile (Trigatti et al., 1999). SR-B1 KO ovaries showed smaller corpora lutea and suboptimal luteal steroid production after ovulation, which agrees with the high expression of SR-B1 in luteal cells (Miranda-Jiménez and Murphy, 2007; Jiménez et al., 2010). Despite this endocrine disorder, SR-B1 KO animals undergo unaltered estrus cycles. They ovulate normally, both naturally and after superovulation, so insufficient hormone synthesis cannot explain SR-B1 KO female infertility (Trigatti et al., 1999). In the first paper reporting the reproductive phenotype of SR-B1 KO females, the authors described that around 60% of the early embryos produced by mating with WT males showed an abnormal, non-refractile morphology, “reminiscent of that seen in embryos mechanically damaged during pronuclear injection” (Trigatti et al., 1999). Those embryos did not develop further and were suspected to be dead. The eggs harvested after superovulation of SR-B1 KO females also showed compromised viability, although in a lower proportion (around 30%). Unexpectedly, SR-B1 was not detected in oocytes.

**TABLE 1:** Fertility and cholesterol levels in plasma and eggs from genetically modified mice with disruptions in HDL metabolism.

Genetic modification	Treatment	TC (mg/dl)	TC (%WT)	HDL-C (mg/dl)	HDL-C (%WT)	HDL UC:TC (ratio) [times vs WT]	HDL size	Oocyte UC fluorescence (%WT)	Fertility	Refs
**SR-B1 KO**	none	126 – 210	172 – 200	107	**170**	**High (0.5) [5X]**	Large	**∼150**	**infertile**	(Trigatti et al., 1999; Yesilaltay et al., 2014; Quiroz et al., 2020)
**SR-B1 KO**	Probucol	108	100	∼90	100	Normal (0.22)	Large	90	fertile	(Miettinen et al., 2001)
**SR-B1 KO**	Liver Tg.SR-B1	7	7	n/d	n/d	Low	n/d	n/r	fertile	(Yesilaltay et al., 2006)
**Apoa1 KO**	none	29	30	13	20	High (0.5) [2X]	Large	n/r	fertile	(Williamson et al., 1992; Plump et al., 1997)
**Apoa1/SR-B1 dKO**	none	105	100	∼70	100	n/r	Large	∼130	subfertile	(Miettinen et al., 2001)
**TgCETP/SR-B1 KO**	none	110	100	85	**150**	**High (0.5) [2.8X]**	Normal	n/r	**infertile**	(Hildebrand et al., 2010)
**ABCA1 KO**	none	20	20	2	3	n/r	Small	∼140	subfertile	(Christiansen-Weber et al., 2000; Francone et al., 2003)
**ABCG1 KO**	none	54	100	∼30	100	Normal (0.24)	Normal	n/r	fertile	(Buchmann et al., 2007; Out et al., 2007)
**LCAT KO**	none	33	30	16	18	*High (0.85) [3.5X]	Small	n/r	fertile	(Ng et al., 1997)
**PDZK1**	none	139	178	∼130	∼180	Normal (0.24)	Large	n/r	fertile	(Kocher et al., 2003)

*Caused by a reduction in CE levels and not by an increase in UC levels.

**Abbreviations:** SR-B1, scavenger receptor class B type 1; Liver Tg.SR-B1, Liver-specific SR-B1 transgenic; Apoa1, apolipoprotein A1; TgCETP, transgenic mice expressing human cholesteryl ester transfer protein; ABCA1, ATP-binding cassette A1 transporter; ABCG1, ATP-binding cassette G1 transporter; LCAT, lecithin-cholesterol acyl transferase; PDZK1, PDZ domain-containing protein 1; TC, total cholesterol; HDL-C, high-density lipoprotein cholesterol; UC, unesterified cholesterol.

Two alternative mechanisms were proposed to explain infertility in SR-B1 KO females (Trigatti et al., 1999). The first involved deficiencies in the provision of cholesterol or other HDL-transported lipids to support follicular cell functions due to the lack of SR-B1 in the ovary itself. This possibility was ruled out by a straightforward experiment involving bilateral transplantation of SR-B1 KO ovaries into immunocompromised ovariectomized WT females (Miettinen et al., 2001). Two to three weeks after transplantation, around 80% of the females with SR-B1 negative ovaries mated with WT males delivered healthy heterozygous pups, showing that the lack of SR-B1 in the ovary was not the cause of infertility in SR-B1 KO females. The second potential mechanism for SR-B1 KO female sterility comprised a negative effect on egg viability of large, dysfunctional HDLs circulating in those animals (Trigatti et al., 1999). Existing evidence suggests that structural and functional properties of FF HDL influence follicle and oocyte biology. To test this hypothesis, M. Krieger and his team used different genetic and pharmacologic strategies to modify the structure of circulating HDL in SR-B1 KO females and subsequently examined their fertility by mating them with WT males (Miettinen et al., 2001). They generated ApoA-I/SR-B1 double KO (dKO) females with smaller, less cholesterol-enriched HDL than those in SR-B1 KO females. HDL from ApoA-I-deficient mice had normal size but altered composition: more UC and less CE. After mating them with WT males, around 40% of dKO females became pregnant and delivered healthy pups, supporting the idea that normalization of HDL structure may restore fertility in SR-B1 KO females. As an additional strategy to test this possibility, researchers from the same group treated SR-B1 KO females with the powerful cholesterol-lowering drug probucol (Barnhart et al., 1970; Miettinen et al., 2001). Total cholesterol (TC) levels were reduced by approximately 50% and the UC:TC ratio was normalized in SR-B1 KO females after administration of 0.5% probucol in the diet for 3 weeks. This intervention restored fertility rapidly and completely: as soon as one week after starting probucol administration, females became pregnant and delivered healthy, normal-sized litters. In a subsequent paper, Krieger´s group explored the effect of overexpressing SR-B1 in the liver of SR-B1 KO females on HDL structure and female fertility (Yesilaltay et al., 2006). They found that hepatic SR-BI expression using adenovirus-mediated transient expression and stable transgenesis completely restored female fertility associated with correcting the two main structural defects of SR-B1 KO HDL, their abnormally large size and high UC:TC ratio. Apparently, reducing the proportion of UC in HDL in these animals seemed to be effective at reversing infertility, similarly to the effect in probucol-treated SR-B1 KO females.

In collaboration with M Krieger´s group, we compared the cholesterol content and distribution in WT and SR-B1 KO eggs using filipin, a fluorescent UC-specific probe. Uniform staining with brighter puncta, suggestive of intracellular UC deposits, was observed in eggs from mice of both genotypes (Yesilaltay et al., 2014). However, SR-B1 KO eggs showed higher filipin fluorescence intensity (∼68%), indicative of UC accumulation (Yesilaltay et al., 2014). In addition, ~ 73% of SR-B1 KO eggs showed spontaneous progression beyond MII arrest after superovulation (Yesilaltay et al., 2014). Also, some of them were found to be dead (∼19%), in agreement with previous results (Trigatti et al., 1999). These results, together with our data showing that artificially induced cholesterol excess using MβCD-Chol in mouse WT eggs promoted their spontaneous activation and parthenogenic cleavage, suggest that cholesterol enrichment may be a cause of exit from MII arrest independent of fertilization (Yesilaltay et al., 2014). Furthermore, those results also link cholesterol excess in SR-B1 KO eggs with female infertility in this model.

We recently showed that cholesterol accumulation in SR-B1 KO oocytes starts at the antral stage of follicular development when oocytes begin to be exposed to FF HDL (Quiroz et al., 2020). In that study, FF HDL were detected in the antrum of SR-B1 KO follicles using ApoA-I indirect immunofluorescence. Although the size and composition of FF HDL could not be characterized due to obvious limitations in the volume of mouse FF samples, cholesterol accumulation in antral oocytes from SR-B1 KO mice is consistent with the hypothesis that disruptions in HDL structure and function may affect egg quality and developmental potential (Browne et al., 2008; Browne et al., 2009; Kim et al., 2017a; Jia et al., 2020). Indeed, probucol administration to SR-B1 KO females, a treatment previously shown to lower HDL UC levels and restore fertility in these mice (Miettinen et al., 2001), normalized UC levels in both immature oocytes and ovulated eggs (Quiroz et al., 2020). The normalization of UC cholesterol levels in gametes from SR-B1 KO females was also achieved by incubating cumuli-denuded eggs with HDL purified from WT plasma, suggesting that efflux of excess cholesterol from eggs to functional HDL may help to maintain cholesterol levels within a physiological range in the gamete. In contrast, incubation of HDL purified from SR-B1 KO plasma with WT eggs did not result in evident lipoprotein-to-gamete transfer of UC. Thus, cholesterol excess in eggs from SR-B1 KO females is probably not caused primarily by UC transfer from FF HDL but due to inefficient efflux to abnormal HDL in SR-B1 KO FF. Although this *in vitro* assay using denuded eggs and purified plasma HDL does not recapitulate what occurs in the ovarian follicle precisely, it suggests that FF HDL may play a key role in cholesterol efflux to maintain cholesterol homeostasis in oocytes. The possibility that the fertilizing ability and developmental potential of SR-B1 KO eggs is restored after exposure to WT HDL is currently being evaluated in our laboratory.

In agreement with the idea that FF HDL participate in UC efflux, ABCA1 and ABCG1 transporters were detected both at the mRNA and protein levels (by immunofluorescence) in eggs (Quiroz et al., 2020). A strong signal for ABCA1 was observed at the egg surface. In addition, we also observed that ABCA1 KO eggs stained with filipin showed ∼30% higher fluorescent levels than WT eggs, suggesting that ABCA1 plays a role in mediating cholesterol efflux from eggs to HDL. Human FF HDL are mostly small and contain low UC content, biochemical features that favor the affinity of this HDL subclass with ABCA1 - and not with ABCG1 (Jaspard et al., 1996; Jaspard et al., 1997). This evidence, together with the fact that ABCG1 KO females are completely fertile (Kennedy et al., 2005), suggests a less significant function of this transporter in egg cholesterol efflux. ABCA1 KO females show approximately 80% reduction in total plasma cholesterol, and HDL with almost no cholesterol and an abnormal phospholipid composition (McNeish et al., 2000; Francone et al., 2003). These females have reduced fertility, with lower pregnancy rates and smaller litter sizes (Christiansen-Weber et al., 2000). Two independent abnormalities seem to combine in these animals. First, their placenta is malformed, so fetuses develop with severe growth retardation, and almost 50% of pups die *in utero* or before weaning. This altered development seems to be explained by a reduction in ovarian steroids: both estrogen and progesterone levels are ∼50% lower in pregnant ABCA1 KO when compared to WT females (Christiansen-Weber et al., 2000). Second, pregnancy rates after mating in ABCA1 KO females are around 45%, suggesting an additional defect in one or more events before pregnancy or placenta development, e.g., gamete production, fertilization, and activation. We recently reported that ∼20% of eggs retrieved from the oviducts of ABCA1 superovulated females appear dead, with a morphology reminiscent of that observed in SR-B1 KO eggs (Quiroz et al., 2020). Although characteristics of ABCA1 KO mice mentioned here suggest that the mechanism explaining the infertility is complex, our recent evidence indicates that the absence of ABCA1 in KO eggs affects their cholesterol homeostasis. Altogether, the phenotype observed in eggs from SR-B1 and ABCA1 KO mice supports the hypothesis that FF HDL and ABC transporters in oocytes are part of a cholesterol homeostatic mechanism preventing UC cholesterol accumulation in oocytes that may be relevant for fertility.

The role of UC as a pathogenic player in female mouse infertility was also suggested by evidence from two genetically modified mouse models with altered HDL metabolism. In one of these models, the PDZ domain containing 1 (PDZK1) KO mouse, a normal UC:TC proportion despite abnormal HDL levels does not affect fertility in females (Kocher et al., 2003). PDZK1 is a multi-PDZ domain containing adaptor protein that interacts with SR-B1 and stabilizes this receptor in a tissue-specific and post-translational manner (Trigatti, 2017). The expression of PDZK1 in the liver is necessary for the stability of SR-B1 in this organ. PDZK1 deficient mice show normal SR-B1 content in ovaries and other steroidogenic tissues, but a very low hepatic SR-B1 levels (5% vs. WT controls) (Kocher et al., 2003). Like SR-B1 KO animals, PDZK1 KO mice have abnormally large, cholesterol-enriched circulating HDL particles. However, the UC:TC ratio in these particles remains almost normal and female PDZK1 KO mice are fertile. An opposite situation is observed in SR-B1 KO mice transgenically expressing Human Cholesteryl Esther Transfer Protein (CETP): TgCETP SR-B1 KO (Hildebrand et al., 2010). CETP is a hydrophobic plasma glycoprotein mainly expressed in human liver, spleen and adipose tissue and naturally absent in mice (Agellon et al., 1991). This enzyme mediates the mobilization of CE between HDL and other lipoproteins, such as VLDL and LDL, so these larger lipoproteins carry around ∼65% of plasma cholesterol in humans (Tall, 1993). Mice and rats do not express functional CETP and cannot transfer CE from HDL to other lipoproteins, so almost all plasma cholesterol in rodents is contained in HDL. TgCETP SR-B1 KO mice were generated to study if CETP-mediated transfer of CE from HDL to larger lipoproteins could replace the inefficient reverse cholesterol transport observed in SR-B1 KO and serve as a compensatory mechanism to prevent atherosclerosis. CETP expression in SR-B1 KO mice significantly reduced HDL particle size and TC levels; however, the UC:TC ratio remained high compared to that in WT females, and TgCETP SR-B1 KO were unable to generate litters over a period of 6 months.

As described previously, UC may become esterified by LCAT within lipoproteins. In LCAT KO mice, plasma esterified cholesterol is significantly reduced whereas plasma UC level remains essentially unchanged, so despite a very high UC:CE ratio, UC levels are normal (Ng et al., 1997). The fact that LCAT KO females are fertile shows that not only the percentage of UC but the net amount of this toxic lipid in lipoproteins is relevant for fertility.

## Conclusion

Cholesterol has been traditionally considered essential for female fertility mainly based on its relevance as a substrate for steroid synthesis in ovarian follicular cells. However, recent work provided evidence showing that oocyte cholesterol homeostasis may also be relevant ensure the developmental potential of eggs.

The small size and low cholesterol content in FF HDL, which apparently permeate from circulation and subsequently undergo remodeling inside the follicle, suggest that these particles play an essential role in maintaining oocyte cholesterol levels within appropriate ranges by participating as acceptors for UC. Indeed, females from mouse models with HDL containing excess UC or undergoing impaired reverse cholesterol transport are infertile. In some of these animals, UC accumulation in oocytes is associated with an increased lability and reduced capacity of generating live embryos.

Infertility affects around 1 in 10 young couples, and as far as 30% of female infertility is idiopathic (of unknown cause) (Sadeghi, 2015). Disorders of lipid metabolism, particularly classic dyslipidemia characterized by high triglycerides and a low HDL-to-LDL cholesterol ratio, are considered independent risk factors of lower fecundability in women (Pugh et al., 2017; Pirnat et al., 2018; Jamro et al., 2019). However, the participation of abnormal HDL in the etiology of infertility in women remains largely unexplored. As far as we are aware of, only one study demonstrated that infertility is associated with disruptions in circulating UC levels. In the Longitudinal Investigation of Fertility and Environment (LIFE) study, higher serum UC levels were reported in couples who did not become pregnant during the 12-months follow-up period. This association was also observed when female partners were analyzed independently: high UC levels were observed in women showing longer time to pregnancy as an outcome of fertility (Schisterman et al., 2014). Unfortunately, neither the distribution of UC among different lipoproteins nor the oocyte phenotype were reported in that study.

**FIGURE 1:**
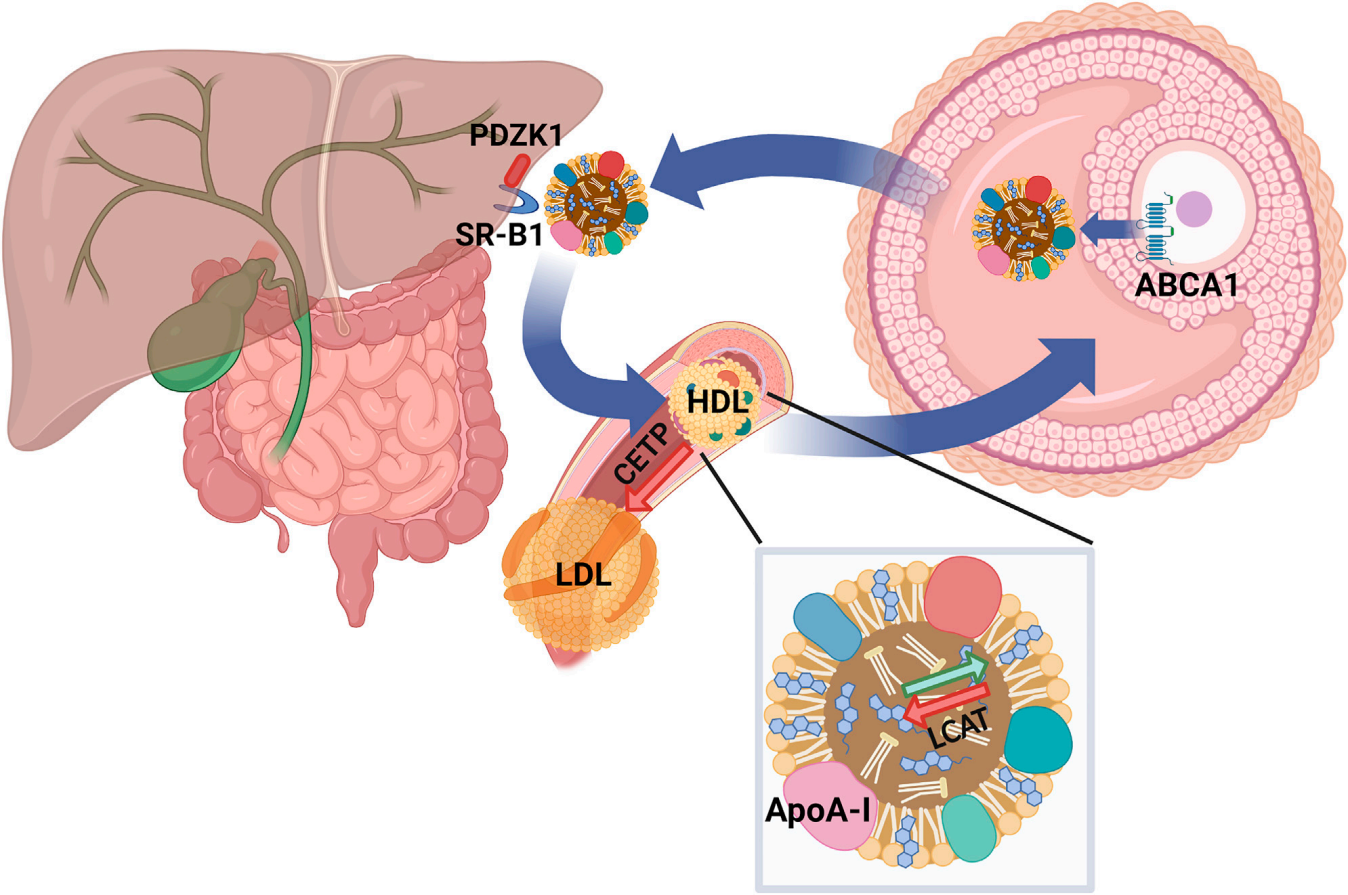
Working model. We propose a homeostatic mechanism in the ovary in which FF HDL ensure adequate cholesterol levels in the egg by removing excess cholesterol from the oocyte during the antral stage of follicular development. SR-B1: Scavenger Receptor Class B Type 1; ABCA1: ATP-Binding Cassette transporter A1; CETP: Cholesteryl Esther Transfer Protein; LCAT: Lecithin Cholesterol Acyl Transferase; PDZK1: PDZ domain containing 1. Created with BioRender.com.

Our current working model (Figure 1) proposes that FF HDL are part of a homeostatic mechanism that ensures adequate cholesterol levels in developing oocytes and eggs by removing excess cholesterol from the PM. Deficient reverse cholesterol transport by abnormal FF HDL results in UC excess in the egg, an alteration that can interfere with biological events modulating oocyte maturation, meiosis arrest and egg activation after fertilization. Thus, alterations in HDL cholesterol metabolism may impact FF HDL function and result in infertility due to egg dysfunction and lability.

## References

[B1] Acton S. Rigotti A. Landschulz K. T. Xu S. Hobbs H. H. Krieger M. (1996). Identification of Scavenger Receptor SR-BI as a High Density Lipoprotein Receptor. Science 271 (5248), 518–520. 10.1126/science.271.5248.518 8560269

[B2] Agellon L. B. Walsh A. Hayek T. Moulin P. Jiang X. C. Shelanski S. A. (1991). Reduced High Density Lipoprotein Cholesterol in Human Cholesteryl Ester Transfer Protein Transgenic Mice. J. Biol. Chem. 266 (17), 10796–10801. 10.1016/s0021-9258(18)99088-5 2040599

[B3] Anderson A. Campo A. Fulton E. Corwin A. Jerome W. G. O'Connor M. S. (2020). 7-Ketocholesterol in Disease and Aging. Redox Biol. 29, 101380. 10.1016/j.redox.2019.101380 31926618 PMC6926354

[B4] Anderson E. Albertini D. F. (1976). Gap Junctions between the Oocyte and Companion Follicle Cells in the Mammalian Ovary. J. Cell. Biol. 71 (2), 680–686. 10.1083/jcb.71.2.680 825522 PMC2109751

[B5] Argov N. Moallem U. Sklan D. (2004). Lipid Transport in the Developing Bovine Follicle: Messenger RNA Expression Increases for Selective Uptake Receptors and Decreases for Endocytosis Receptors. Biol. Reprod. 71 (2), 479–485. 10.1095/biolreprod.104.028092 15056566

[B6] Atalla H. K. Arıcı R. Yagcıoglu S. Eser A. Ersoy N. (2018). Effect of Cholesterol Loaded Methyl-β-Cyclodextrin on Ovine Oocytes during Chilling and Vitrification. Rev. médecine vétérinaire 169, 241–246.

[B7] Barnhart J. W. Sefranka J. A. McIntosh D. D. (1970). Hypocholesterolemic Effect of 4,4′-(Isopropylidenedithio)-Bis(2,6-Di-T-Butylphenol) (Probucol). Am. J. Clin. Nutr. 23 (9), 1229–1233. 10.1093/ajcn/23.9.1229 5450842

[B8] Bellanti F. Villani R. Tamborra R. Blonda M. Iannelli G. di Bello G. (2018). Synergistic Interaction of Fatty Acids and Oxysterols Impairs Mitochondrial Function and Limits Liver Adaptation during Nafld Progression. Redox Biol. 15, 86–96. 10.1016/j.redox.2017.11.016 29220698 PMC5725223

[B9] Bełtowski J. Semczuk A. (2010). Liver X Receptor (LXR) and the Reproductive System-Aa Potential Novel Target for Therapeutic Intervention. Pharmacol. Rep. 62 (1), 15–27. 10.1016/s1734-1140(10)70239-5 20360612

[B10] Bernhardt M. L. Stein P. Carvacho I. Krapp C. Ardestani G. Mehregan A. (2018). TRPM7 and CaV3.2 Channels Mediate Ca^2^+ Influx Required for Egg Activation at Fertilization. Proc. Natl. Acad. Sci. U. S. A. 115 (44), E10370–e10378. 10.1073/pnas.1810422115 30322909 PMC6217414

[B11] Bhonagiri P. Pattar G. R. Habegger K. M. McCarthy A. M. Tackett L. Elmendorf J. S. (2011). Evidence Coupling Increased Hexosamine Biosynthesis Pathway Activity to Membrane Cholesterol Toxicity and Cortical Filamentous Actin Derangement Contributing to Cellular Insulin Resistance†. Endocrinology 152 (9), 3373–3384. 10.1210/en.2011-1295 21712361 PMC3159786

[B12] Bianchi E. Doe B. Goulding D. Wright G. J. (2014). Juno Is the Egg Izumo Receptor and Is Essential for Mammalian Fertilization. Nature 508 (7497), 483–487. 10.1038/nature13203 24739963 PMC3998876

[B13] Blackett P. R. McConathy W. J. (1982). Comparison of Lipids and Apolipoproteins in Amniotic Fluid, Neonatal Urine, and Cord Serum. Ann. Clin. Lab. Sci. 12 (4), 288–295. 6814342

[B14] Bradley J. Swann K. (2019). Mitochondria and Lipid Metabolism in Mammalian Oocytes and Early Embryos. Int. J. Dev. Biol. 63 (3-4-5), 93–103. 10.1387/ijdb.180355ks 31058306

[B15] Brantmeier S. A. Grummer R. R. Ax R. L. (1987). Concentrations of High Density Lipoproteins Vary Among Follicular Sizes in the Bovine. J. Dairy Sci. 70 (10), 2145–2149. 10.3168/jds.s0022-0302(87)80266-7 3680733

[B16] Brooks-Wilson A. Marcil M. Clee S. M. Zhang L.-H. Roomp K. van Dam M. (1999). Mutations in ABC1 in Tangier Disease and Familial High-Density Lipoprotein Deficiency. Nat. Genet. 22 (4), 336–345. 10.1038/11905 10431236

[B17] Browne R. W. Bloom M. S. Shelly W. B. Ocque A. J. Huddleston H. G. Fujimoto V. Y. (2009). Follicular Fluid High Density Lipoprotein-Associated Micronutrient Levels Are Associated with Embryo Fragmentation during IVF. J. Assist. Reprod. Genet. 26 (11-12), 557–560. 10.1007/s10815-009-9367-x 19921421 PMC2799562

[B18] Browne R. W. Shelly W. B. Bloom M. S. Ocque A. J. Sandler J. R. Huddleston H. G. (2008). Distributions of High-Density Lipoprotein Particle Components in Human Follicular Fluid and Sera and Their Associations with Embryo Morphology Parameters during IVF. Hum. Reprod. 23 (8), 1884–1894. 10.1093/humrep/den183 18487218

[B19] Buchmann J. Meyer C. Neschen S. Augustin R. Schmolz K. Kluge R. (2007). Ablation of the Cholesterol Transporter Adenosine Triphosphate-Binding Cassette Transporter G1 Reduces Adipose Cell Size and Protects against Diet-Induced Obesity. Endocrinology 148 (4), 1561–1573. 10.1210/en.2006-1244 17194745

[B20] Buschiazzo J. Bonini I. C. Alonso T. S. (2008). Inhibition of Bufo Arenarum Oocyte Maturation Induced by Cholesterol Depletion by Methyl-β-Cyclodextrin. Role of Low-Density Caveolae-Like Membranes. Biochimica Biophysica Acta (BBA) - Biomembr. 1778 (6), 1398–1406. 10.1016/j.bbamem.2008.03.004 18395513

[B21] Buschiazzo J. Ialy-Radio C. Auer J. Wolf J.-P. Serres C. Lefèvre B. (2013). Cholesterol Depletion Disorganizes Oocyte Membrane Rafts Altering Mouse Fertilization. PLoS One 8 (4), e62919. 10.1371/journal.pone.0062919 23638166 PMC3636221

[B22] Buschiazzo J. Ríos G. L. Canizo J. R. Antollini S. S. Alberio R. H. (2017). Free Cholesterol and Cholesterol Esters in Bovine Oocytes: Implications in Survival and Membrane Raft Organization after Cryopreservation. PLoS One 12 (7), e0180451. 10.1371/journal.pone.0180451 28686720 PMC5501518

[B23] Chang S. C. S. Jones J. D. Ellefson R. D. Ryan R. J. (1976). The Porcine Ovarian Follicle: I. Selected Chemical Analysis of Follicular Fluid at Different Developmental Stages1. Biol. Reproduction 15 (3), 321–328. 10.1095/biolreprod15.3.321 183842

[B24] Chang T. Y. Chang C. C. Y. Cheng D. (1997). Acyl-Coenzyme A:Cholesterol Acyltransferase. Annu. Rev. Biochem. 66, 613–638. 10.1146/annurev.biochem.66.1.613 9242919

[B25] Chang X.-L. Liu L. Wang N. Chen Z.-J. Zhang C. (2017). The Function of High-Density Lipoprotein and Low-Density Lipoprotein in the Maintenance of Mouse Ovarian Steroid Balance†. Biol. Reprod. 97 (6), 862–872. 10.1093/biolre/iox134 29092018

[B26] Chen X. Dong H. Cheng M. Wang Q. Jin Y. (2019). Addition of Cholesterol Loaded Cyclodextrin Prior to GV-Phase Vitrification Improves the Quality of Mature Porcine Oocytes *In Vitro* . Cryobiology 90, 54–62. 10.1016/j.cryobiol.2019.08.006 31446003

[B27] Cherian-Shaw M. Puttabyatappa M. Greason E. Rodriguez A. VandeVoort C. A. Chaffin C. L. (2009). Expression of Scavenger Receptor-BI and Low-Density Lipoprotein Receptor and Differential Use of Lipoproteins to Support Early Steroidogenesis in Luteinizing Macaque Granulosa Cells. Endocrinology 150 (2), 957–965. 10.1210/en.2008-0619 18832102 PMC2646541

[B28] Choi D.-H. Lee W.-S. Won M. Park M. Park H.-O. Kim E. (2010). The Apolipoprotein A-I Level Is Downregulated in the Granulosa Cells of Patients with Polycystic Ovary Syndrome and Affects Steroidogenesis. J. Proteome Res. 9 (9), 4329–4336. 10.1021/pr100008e 20426491

[B29] Christiansen-Weber T. A. Voland J. R. Wu Y. Ngo K. Roland B. L. Nguyen S. (2000). Functional Loss of ABCA1 in Mice Causes Severe Placental Malformation, Aberrant Lipid Distribution, and Kidney Glomerulonephritis as Well as High-Density Lipoprotein Cholesterol Deficiency. Am. J. Pathology 157 (3), 1017–1029. 10.1016/s0002-9440(10)64614-7 PMC1885686 10980140

[B30] Cortes V. A. Busso D. Mardones P. Maiz A. Arteaga A. Nervi F. (2013). Advances in the Physiological and Pathological Implications of Cholesterol. Biol. Rev. Camb Philos. Soc. 88 (4), 825–843. 10.1111/brv.12025 23445165

[B31] Deng C.-F. Zhu N. Zhao T.-J. Li H.-F. Gu J. Liao D.-F. (2022). Involvement of LDL and Ox-LDL in Cancer Development and its Therapeutical Potential. Front. Oncol. 12, 803473. 10.3389/fonc.2022.803473 35251975 PMC8889620

[B32] Duewell P. Kono H. Rayner K. J. Sirois C. M. Vladimer G. Bauernfeind F. G. (2010). NLRP3 Inflammasomes Are Required for Atherogenesis and Activated by Cholesterol Crystals. Nature 464 (7293), 1357–1361. 10.1038/nature08938 20428172 PMC2946640

[B33] Fahiminiya S. Gérard N. (2010). Follicular Fluid in Mammals. Gynecol. Obstet. Fertil. 38 (6), 402–404. 10.1016/j.gyobfe.2010.04.010 20576551

[B34] Favor O. K. Pestka J. J. Bates M. A. Lee K. S. S. (2021). Centrality of Myeloid-Lineage Phagocytes in Particle-Triggered Inflammation and Autoimmunity. Front. Toxicol. 3, 777768. 10.3389/ftox.2021.777768 35295146 PMC8915915

[B35] Francone O. L. Subbaiah P. V. van Tol A. Royer L. Haghpassand M. (2003). Abnormal Phospholipid Composition Impairs HDL Biogenesis and Maturation in Mice Lacking Abca1. Biochemistry 42 (28), 8569–8578. 10.1021/bi034540v 12859204

[B36] Frohlich J. McLeod R. Hon K. (1982). Lecithin: Cholesterol Acyl Transferase (LCAT). Clin. Biochem. 15 (6), 269–278. 10.1016/s0009-9120(82)96758-3 6762928

[B37] Fujimoto V. Y. Kane J. P. Ishida B. Y. Bloom M. S. Browne R. W. (2010). High-Density Lipoprotein Metabolism and the Human Embryo. Hum. Reprod. Update 16 (1), 20–38. 10.1093/humupd/dmp029 19700490

[B38] Galle-Treger L. Moreau M. Ballaire R. Poupel L. Huby T. Sasso E. (2020). Targeted Invalidation of SR-B1 in Macrophages Reduces Macrophage Apoptosis and Accelerates Atherosclerosis. Cardiovasc. Res. 116 (3), 554–565. 10.1093/cvr/cvz138 31119270

[B39] Garrido N. (2000). Follicular Hormonal Environment and Embryo Quality in Women with Endometriosis. Hum. Reprod. Update 6 (1), 67–74. 10.1093/humupd/6.1.67 10711831

[B40] Gautier T. Becker S. Drouineaud V. Ménétrier F. Sagot P. Nofer J.-R. (2010). Human Luteinized Granulosa Cells Secrete apoB100-Containing Lipoproteins. J. Lipid Res. 51 (8), 2245–2252. 10.1194/jlr.m005181 20407020 PMC2903810

[B41] Geltinger F. Schartel L. Wiederstein M. Tevini J. Aigner E. Felder T. K. (2020). Friend or Foe: Lipid Droplets as Organelles for Protein and Lipid Storage in Cellular Stress Response. Aging Dis. Mol. 25 (21), 5053. 10.3390/molecules25215053 PMC7663626 33143278

[B42] Gonzalez M. B. Robker R. L. Rose R. D. (2022). Obesity and Oocyte Quality: Significant Implications for ART and Emerging Mechanistic Insights. Biol. Reprod. 106 (2), 338–350. 10.1093/biolre/ioab228 34918035

[B43] Gordo A. C. Rodrigues P. Kurokawa M. Jellerette T. Exley G. E. Warner C. (2002). Intracellular Calcium Oscillations Signal Apoptosis Rather Than Activation in *In Vitro* Aged Mouse Eggs1. Biol. Reprod. 66 (6), 1828–1837. 10.1095/biolreprod66.6.1828 12021069

[B44] Hao T. Zhang P. Hao H. Du W. Pang Y. Zhao S. (2021). The Combination Treatment of Cholesterol‐Loaded Methyl‐β‐Cyclodextrin and Methyl‐β‐Cyclodextrin Significantly Improves the Fertilization Capacity of Vitrified Bovine Oocytes by Protecting Fertilization Protein JUNO. Reprod. Dom. Anim. 56 (3), 519–530. 10.1111/rda.13890 33405303

[B45] Hermann M. Lindstedt K. A. Foisner R. Mörwald S. Mahon M. G. Wandl R. (1998). Apolipoprotein A‐I Production by Chicken Granulosa Cells. FASEB J. 12 (10), 897–903. 10.1096/fasebj.12.10.897 9657529

[B46] Hildebrand R. B. Lammers B. Meurs I. Korporaal S. J. A. De Haan W. Zhao Y. (2010). Restoration of High-Density Lipoprotein Levels by Cholesteryl Ester Transfer Protein Expression in Scavenger Receptor Class B Type I (SR-BI) Knockout Mice Does Not Normalize Pathologies Associated with SR-BI Deficiency. Atvb 30 (7), 1439–1445. 10.1161/atvbaha.110.205153 20431066

[B47] Hoekstra M. Van Berkel T. J. Van Eck M. (2010). Scavenger Receptor BI: a Multi-Purpose Player in Cholesterol and Steroid Metabolism. World J. Gastroenterol. 16 (47), 5916–5924. 10.3748/wjg.v16.i47.5916 21157967 PMC3007109

[B48] Ikonen E. Zhou X. (2021). Cholesterol Transport between Cellular Membranes: A Balancing Act between Interconnected Lipid Fluxes. Dev. Cell. 56 (10), 1430–1436. 10.1016/j.devcel.2021.04.025 34004151

[B49] Jamro E. L. Bloom M. S. Browne R. W. Kim K. Greenwood E. A. Fujimoto V. Y. (2019). Preconception Serum Lipids and Lipophilic Micronutrient Levels Are Associated with Live Birth Rates after IVF. Reprod. Biomed. Online 39 (4), 665–673. 10.1016/j.rbmo.2019.06.004 31405720 PMC6779515

[B50] Jaspard B. Fournier N. Vieitez G. Atger V. Barbaras R. Vieu C. (1997). Structural and Functional Comparison of HDL from Homologous Human Plasma and Follicular Fluid. Atvb 17 (8), 1605–1613. 10.1161/01.atv.17.8.1605 9301642

[B51] Jaspard B. Xavier Collet,* fnm. Barbaras R. Manent J. Claude Vieu fnm. Parinaud J. (1996). Biochemical Characterization of Pre-β1 High-Density Lipoprotein from Human Ovarian Follicular Fluid: Evidence for the Presence of a Lipid Core,. Biochemistry 35 (5), 1352–1357. 10.1021/bi950938i 8634263

[B52] Jia C. Nagy R. A. Homminga I. Hoek A. Tietge U. J. F. (2020). The Anti-Inflammatory Function of Follicular Fluid HDL and Outcome of Modified Natural Cycle *In Vitro* Fertilization†. Biol. Reprod. 103 (1), 7–9. 10.1093/biolre/ioaa061 32333006 PMC7313252

[B53] Jiménez L. M. Binelli M. Bertolin K. Pelletier R. M. Murphy B. D. (2010). Scavenger Receptor-B1 and Luteal Function in Mice. J. Lipid Res. 51 (8), 2362–2371. 10.1194/jlr.m006973 20404351 PMC2903804

[B54] Jonas A. Phillips M. C. (2008). “Lipoprotein Structure,” in Biochemistry of Lipids, Lipoproteins and Membranes. Editors Vance D. E. Vance J. E.. Fifth Edition (San Diego: Elsevier), 485–506. 10.1016/b978-044453219-0.50019-2

[B55] Kennedy M. A. Barrera G. C. Nakamura K. Baldán Á. Tarr P. Fishbein M. C. (2005). ABCG1 Has a Critical Role in Mediating Cholesterol Efflux to HDL and Preventing Cellular Lipid Accumulation. Cell. Metab. 1 (2), 121–131. 10.1016/j.cmet.2005.01.002 16054053

[B56] Kim K. Bloom M. S. Browne R. W. Bell E. M. Yucel R. M. Fujimoto V. Y. (2017). Associations between Follicular Fluid High Density Lipoprotein Particle Components and Embryo Quality Among *In Vitro* Fertilization Patients. J. Assist. Reprod. Genet. 34 (1), 1–10. 10.1007/s10815-016-0826-x PMC5237431 27900613

[B57] Kim K. Bloom M. S. Fujimoto V. Y. Browne R. W. (2017). Associations between PON1 Enzyme Activities in Human Ovarian Follicular Fluid and Serum Specimens. PLoS One 12 (2), e0172193. 10.1371/journal.pone.0172193 28196109 PMC5308615

[B58] Kocher O. Yesilaltay A. Cirovic C. Pal R. Rigotti A. Krieger M. (2003). Targeted Disruption of the PDZK1 Gene in Mice Causes Tissue-Specific Depletion of the High Density Lipoprotein Receptor Scavenger Receptor Class B Type I and Altered Lipoprotein Metabolism. J. Biol. Chem. 278 (52), 52820–52825. 10.1074/jbc.m310482200 14551195

[B59] Kolmakova A. Wang J. Brogan R. Chaffin C. Rodriguez A. (2010). Deficiency of Scavenger Receptor Class B Type I Negatively Affects Progesterone Secretion in Human Granulosa Cells. Endocrinology 151 (11), 5519–5527. 10.1210/en.2010-0347 20844007 PMC3208332

[B60] Korac B. Kalezic A. Pekovic-Vaughan V. Korac A. Jankovic A. (2021). Redox Changes in Obesity, Metabolic Syndrome, and Diabetes. Redox Biol. 42, 101887. 10.1016/j.redox.2021.101887 33579666 PMC8113039

[B61] Lai W.-A. Yeh Y.-T. Lee M.-T. Wu L.-S. Ke F.-C. Hwang (黃娟娟 J.-J. (2013). Ovarian Granulosa Cells Utilize Scavenger Receptor SR-BI to Evade Cellular Cholesterol Homeostatic Control for Steroid Synthesis. J. Lipid Res. 54 (2), 365–378. 10.1194/jlr.m030239 23197320 PMC3588866

[B62] Lakk M. Hoffmann G. F. Gorusupudi A. Enyong E. Lin A. Bernstein P. S. (2021). Membrane Cholesterol Regulates TRPV4 Function, Cytoskeletal Expression, and the Cellular Response to Tension. J. Lipid Res. 62, 100145. 10.1016/j.jlr.2021.100145 34710431 PMC8633027

[B63] Lange Y. Ye J. Steck T. L. (2014). Essentially All Excess Fibroblast Cholesterol Moves from Plasma Membranes to Intracellular Compartments. PLOS ONE 9 (7), e98482. 10.1371/journal.pone.0098482 25014655 PMC4094430

[B64] Le Goff D. (1994). Follicular Fluid Lipoproteins in the Mare: Evaluation of HDL Transfer from Plasma to Follicular Fluid. Biochimica Biophysica Acta (BBA) - Lipids Lipid Metabolism 1210 (2), 226–232. 10.1016/0005-2760(94)90125-2 8280774

[B65] Li L. Zhu S. Shu W. Guo Y. Guan Y. Zeng J. (2020). Characterization of Metabolic Patterns in Mouse Oocytes during Meiotic Maturation. Mol. Cell. 80 (3), 525–540. e9. 10.1016/j.molcel.2020.09.022 33068521 PMC8034554

[B66] Liu J. Gillard B. K. Yelamanchili D. Gotto A. M. Rosales C. Pownall H. J. (2021). High Free Cholesterol Bioavailability Drives the Tissue Pathologies in Scarb1^-/-^ Mice. Arterioscler. Thromb. Vasc. Biol. 41 (10), e453–e467. 10.1161/ATVBAHA.121.316535 34380332 PMC8458258

[B67] Luo J. Yang H. Song B.-L. (2020). Mechanisms and Regulation of Cholesterol Homeostasis. Nat. Rev. Mol. Cell. Biol. 21 (4), 225–245. 10.1038/s41580-019-0190-7 31848472

[B68] Mahley R. W. (2016). Central Nervous System Lipoproteins. Atvb 36 (7), 1305–1315. 10.1161/atvbaha.116.307023 PMC4942259 27174096

[B69] Marco-Jimenez F. Jimenez-Trigos E. Lavara R. Vicente J. S. (2014). Use of Cyclodextrins to Increase Cytoplasmic Cholesterol in Rabbit Embryos and Their Impact on Live Kits Derived from Vitrified Embryos. Cryo Lett. 35 (4), 320–326. 25282500

[B70] Marí M. Caballero F Colell A Morales A Caballeria J Fernandez A (2006). Mitochondrial Free Cholesterol Loading Sensitizes to TNF- and Fas-Mediated Steatohepatitis. Cell. Metab. 4 (3), 185–198. 10.1016/j.cmet.2006.07.006 16950136

[B71] McNeish J. Aiello R. J. Guyot D. Turi T. Gabel C. Aldinger C. (2000). High Density Lipoprotein Deficiency and Foam Cell Accumulation in Mice with Targeted Disruption of ATP-Binding Cassette Transporter-1. Proc. Natl. Acad. Sci. U.S.A. 97 (8), 4245–4250. 10.1073/pnas.97.8.4245 10760292 PMC18215

[B72] Mehregan A. Ardestani G. Akizawa H. Carvacho I. Fissore R. (2021). Deletion of TRPV3 and Ca_V_3.2 T-Type Channels in Mice Undermines Fertility and Ca^2+^ Homeostasis in Oocytes and Eggs. J. Cell. Sci. 134 (13). 10.1242/jcs.257956 PMC8313860 34313315

[B73] Miettinen H. E. Rayburn H. Krieger M. (2001). Abnormal Lipoprotein Metabolism and Reversible Female Infertility in HDL Receptor (SR-BI)-Deficient Mice. J. Clin. Investig. 108 (11), 1717–1722. 10.1172/jci13288 11733567 PMC200987

[B74] Milhiet P. E. Giocondi M.-C. Le Grimellec C. (2002). Cholesterol Is Not Crucial for the Existence of Microdomains in Kidney Brush-Border Membrane Models. J. Biol. Chem. 277 (2), 875–878. 10.1074/jbc.c100654200 11717303

[B75] Mineo C. (2020). Lipoprotein Receptor Signalling in Atherosclerosis. Cardiovasc Res. 116 (7), 1254–1274. 10.1093/cvr/cvz338 31834409 PMC7243280

[B76] Miranda-Jiménez L. Murphy B. D. (2007). Lipoprotein Receptor Expression during Luteinization of the Ovarian Follicle. Am. J. Physiology-Endocrinology Metabolism 293 (4), E1053–E1061. 10.1152/ajpendo.00554.2006 17698983

[B77] Montero J. Mari M. Colell A. Morales A. Basañez G. Garcia-Ruiz C. (2010). Cholesterol and Peroxidized Cardiolipin in Mitochondrial Membrane Properties, Permeabilization and Cell Death. Biochim. Biophys. Acta 1797 (6-7), 1217–1224. 10.1016/j.bbabio.2010.02.010 20153716 PMC2889134

[B78] Nakanishi T. Tanaka R. Tonai S. Lee J. Y. Yamaoka M. Kawai T. (2021). LH Induces De Novo Cholesterol Biosynthesis *via* SREBP Activation in Granulosa Cells during Ovulation in Female Mice. Endocrinology 162 (11). 10.1210/endocr/bqab166 34431998

[B79] Ng D. S. Francone O. L. Forte T. M. Zhang J. Haghpassand M. Rubin E. M. (1997). Disruption of the Murine Lecithin:Cholesterol Acyltransferase Gene Causes Impairment of Adrenal Lipid Delivery and Up-Regulation of Scavenger Receptor Class B Type I. J. Biol. Chem. 272 (25), 15777–15781. 10.1074/jbc.272.25.15777 9188474

[B80] Nicholson A. M. Ferreira A. (2009). Increased Membrane Cholesterol Might Render Mature Hippocampal Neurons More Susceptible to -Amyloid-Induced Calpain Activation and Tau Toxicity. J. Neurosci. 29 (14), 4640–4651. 10.1523/jneurosci.0862-09.2009 19357288 PMC2705291

[B81] Ouimet M. Marcel Y. L. (2012). Regulation of Lipid Droplet Cholesterol Efflux from Macrophage Foam Cells. Atvb 32 (3), 575–581. 10.1161/atvbaha.111.240705 22207731

[B82] Out R. Hoekstra M. Meurs I. de Vos P. Kuiper J. Van Eck M. (2007). Total Body ABCG1 Expression Protects against Early Atherosclerotic Lesion Development in Mice. Atvb 27 (3), 594–599. 10.1161/01.atv.0000257136.24308.0c 17204665

[B83] Ozil J.-P. Markoulaki S. Toth S. Matson S. Banrezes B. Knott J. G. (2005). Egg Activation Events Are Regulated by the Duration of a Sustained [Ca^2+^]_cyt_ Signal in the Mouse. Dev. Biol. 282 (1), 39–54. 10.1016/j.ydbio.2005.02.035 15936328

[B84] Parinaud J. Perret B. Ribbes H. Chap H. Pontonnier G. Douste-blazy L. (1987). High Density Lipoprotein and Low Density Lipoprotein Utilization by Human Granulosa Cells for Progesterone Synthesis in Serum-Free Culture: Respective Contributions of Free and Esterified Cholesterol. J. Clin. Endocrinol. Metabolism 64 (3), 409–417. 10.1210/jcem-64-3-409 3818885

[B85] Phillips M. C. (2014). Molecular Mechanisms of Cellular Cholesterol Efflux. J. Biol. Chem. 289 (35), 24020–24029. 10.1074/jbc.r114.583658 25074931 PMC4148835

[B86] Pirnat A. DeRoo L. A. Skjærven R. Morken N.-H. (2018). Women's Prepregnancy Lipid Levels and Number of Children: A Norwegian Prospective Population-Based Cohort Study. BMJ Open 8 (6), e021188. 10.1136/bmjopen-2017-021188 PMC6042606 29986867

[B87] Plump A. S. Azrolan N. Odaka H. Wu L. Jiang X. Tall A. (1997). ApoA-I Knockout Mice: Characterization of HDL Metabolism in Homozygotes and Identification of a Post-RNA Mechanism of apoA-I Up-Regulation in Heterozygotes. J. Lipid Res. 38 (5), 1033–1047. 10.1016/s0022-2275(20)37227-8 9186920

[B88] Pugh S. J. Schisterman E. F. Browne R. W. Lynch A. M. Mumford S. L. Perkins N. J. (2017). Preconception Maternal Lipoprotein Levels in Relation to Fecundability. Hum. Reprod. 32 (5), 1055–1063. 10.1093/humrep/dex052 28333301 PMC6075456

[B89] Quiroz A. Molina P. Santander N. Gallardo D. Rigotti A. Busso D. (2020). Ovarian Cholesterol Efflux: ATP-Binding Cassette Transporters and Follicular Fluid HDL Regulate Cholesterol Content in Mouse Oocytes†. Biol. Reprod. 102 (2), 348–361. 10.1093/biolre/ioz159 31423535

[B90] Rajamäki K. Lappalainen J. Öörni K. Välimäki E. Matikainen S. Kovanen P. T. (2010). Cholesterol Crystals Activate the NLRP3 Inflammasome in Human Macrophages: A Novel Link between Cholesterol Metabolism and Inflammation. PLoS One 5 (7), e11765. 10.1371/journal.pone.0011765 20668705 PMC2909263

[B91] Revelli A. Piane L. D. Casano S. Molinari E. Massobrio M. Rinaudo P. (2009). Follicular Fluid Content and Oocyte Quality: From Single Biochemical Markers to Metabolomics. Reprod. Biol. Endocrinol. 7, 40. 10.1186/1477-7827-7-40 19413899 PMC2685803

[B92] Rigotti A. Miettinen H. E. Krieger M. (2003). The Role of the High-Density Lipoprotein Receptor SR-BI in the Lipid Metabolism of Endocrine and Other Tissues. Endocr. Rev. 24 (3), 357–387. 10.1210/er.2001-0037 12788804

[B93] Rigotti A. Trigatti B. L. Penman M. Rayburn H. Herz J. Krieger M. (1997). A Targeted Mutation in the Murine Gene Encoding the High Density Lipoprotein (HDL) Receptor Scavenger Receptor Class B Type I Reveals its Key Role in HDL Metabolism. Proc. Natl. Acad. Sci. U.S.A. 94 (23), 12610–12615. 10.1073/pnas.94.23.12610 9356497 PMC25055

[B94] Rosenbaum T. Benítez-Angeles M. Sánchez-Hernández R. Morales-Lázaro S. L. Hiriart M. Morales-Buenrostro L. E. (2020). TRPV4: A Physio and Pathophysiologically Significant Ion Channel. Int. J. Mol. Sci. 21 (11). 10.3390/ijms21113837 PMC7312103 32481620

[B95] Sadeghi M. R. (2015). Unexplained Infertility, the Controversial Matter in Management of Infertile Couples. J. Reprod. Infertil. 16 (1), 1–2. 25717428 PMC4322174

[B96] Salter A. M. Brindley D. N. (1988). The Biochemistry of Lipoproteins. J. Inherit. Metab. Dis. 11 (Suppl. 1), 4–17. 10.1007/978-94-009-1259-5_1 3141685

[B97] Sato N. Kawamura K. Fukuda J. Honda Y. Sato T. Tanikawa H. (2003). Expression of LDL Receptor and Uptake of LDL in Mouse Preimplantation Embryos. Mol. Cell. Endocrinol. 202 (1-2), 191–194. 10.1016/s0303-7207(03)00082-0 12770750

[B98] Schisterman E. F. Mumford S. L. Browne R. W. Barr D. B. Chen Z. Louis G. M. B. (2014). Lipid Concentrations and Couple Fecundity: The LIFE Study. J. Clin. Endocrinol. Metab. 99 (8), 2786–2794. 10.1210/jc.2013-3936 24846535 PMC4121020

[B99] Schweigert F. J. (2003). Concentrations of Carotenoids, Retinol and Alpha-Tocopherol in Plasma and Follicular Fluid of Women Undergoing IVF. Hum. Reprod. 18 (6), 1259–1264. 10.1093/humrep/deg249 12773456

[B100] Seong J. Wang Y. Kinoshita T. Maeda Y. (2013). Implications of Lipid Moiety in Oligomerization and Immunoreactivities of GPI-Anchored Proteins. J. Lipid Res. 54 (4), 1077–1091. 10.1194/jlr.m034421 23378600 PMC3605984

[B101] Shalgi R. Kraicer P. Rimon A. Pinto M. Soferman N. (1973). Proteins of Human Follicular Fluid: The Blood-Follicle Barrier**Presented at the Annual Conference of the Society for the Study of Fertility, Reading, England, July 18-22, 1972. Fertil. Steril. 24 (6), 429–434. 10.1016/s0015-0282(16)39730-8 4122802

[B102] Shim Y.-H. Chun J. H. Lee E.-Y. Paik Y.-K. (2002). Role of Cholesterol in Germ-Line Development ofCaenorhabditis Elegans. Mol. Reprod. Dev. 61 (3), 358–366. 10.1002/mrd.10099 11835581

[B103] Shu F. Chen J. Ma X. Fan Y. Yu L. Zheng W. (2018). Cholesterol Crystal-Mediated Inflammation Is Driven by Plasma Membrane Destabilization. Front. Immunol. 9, 1163. 10.3389/fimmu.2018.01163 29896195 PMC5986904

[B104] Silvestris E. de Pergola G. Rosania R. Loverro G. (2018). Obesity as Disruptor of the Female Fertility. Reprod. Biol. Endocrinol. 16 (1), 22. 10.1186/s12958-018-0336-z 29523133 PMC5845358

[B105] Simpson E. R. Rochelle D. B. Carr B. R. MacDonald P. C. (1980). Plasma Lipoproteins in Follicular Fluid of Human Ovaries. J. Clin. Endocrinol. Metabolism 51 (6), 1469–1471. 10.1210/jcem-51-6-1469 7440708

[B106] Skoblina M. N. Shmerling Z. G. Kondratieva O. T. (1981). Cholesterol-Induced *In Vitro* Maturation of Oocytes of *Acipenser stellatus*, *Xenopus laevis*, and *Rana temporaria* . General Comp. Endocrinol. 44 (4), 470–475. 10.1016/0016-6480(81)90334-8 6973502

[B107] Su Y.-Q. Sugiura K. Wigglesworth K. O'Brien M. J. Affourtit J. P. Pangas S. A. (2008). Oocyte Regulation of Metabolic Cooperativity between Mouse Cumulus Cells and Oocytes: BMP15 and GDF9 Control Cholesterol Biosynthesis in Cumulus Cells. Development 135 (1), 111–121. 10.1242/dev.009068 18045843

[B108] Suchanek E. Šimunić V. Kopjar B. Maćaš E. Grizelj V. Salzer B. (1988). Lipid and Lipoprotein Contents of Human Follicular Fluid. J. Clin. Chem. Clin. Biochem. 26 (9), 543–547. 10.1515/cclm.1988.26.9.543 3199076

[B109] Tabas I. (1997). Free Cholesterol-Induced Cytotoxicity. Trends Cardiovasc. Med. 7 (7), 256–263. 10.1016/s1050-1738(97)00086-8 21235894

[B110] Tall A. (1993). Plasma Cholesteryl Ester Transfer Protein. J. Lipid Res. 34 (8), 1255–1274. 10.1016/s0022-2275(20)36957-1 8409761

[B111] Trigatti B. L. Krieger M. Rigotti A. (2003). Influence of the HDL Receptor SR-BI on Lipoprotein Metabolism and Atherosclerosis. Atvb 23 (10), 1732–1738. 10.1161/01.atv.0000091363.28501.84 12920050

[B112] Trigatti B. L. (2017). SR-B1 and PDZK1. Curr. Opin. Lipidol. 28 (2), 201–208. 10.1097/mol.0000000000000396 28134663

[B113] Trigatti B. Rayburn H. Viñals M. Braun A. Miettinen H. Penman M. (1999). Influence of the High Density Lipoprotein Receptor SR-BI on Reproductive and Cardiovascular Pathophysiology. Proc. Natl. Acad. Sci. U.S.A. 96 (16), 9322–9327. 10.1073/pnas.96.16.9322 10430941 PMC17781

[B114] van Montfoort A. P. A. Plösch T. Hoek A. Tietge U. J. F. (2014). Impact of Maternal Cholesterol Metabolism on Ovarian Follicle Development and Fertility. J. Reproductive Immunol. 104-105, 32–36. 10.1016/j.jri.2014.04.003 24933118

[B115] Vejux A. Abed-Vieillard D. Hajji K. Zarrouk A. Mackrill J. J. Ghosh S. (2020). 7-Ketocholesterol and 7β-Hydroxycholesterol: *In Vitro* and Animal Models Used to Characterize Their Activities and to Identify Molecules Preventing Their Toxicity. Biochem. Pharmacol. 173, 113648. 10.1016/j.bcp.2019.113648 31586589

[B116] Velasco M. Alexander C. King J. Zhao Y. Garcia J. Rodriguez A. (2006). Association of Lower Plasma Estradiol Levels and Low Expression of Scavenger Receptor Class B, Type I in Infertile Women. Fertil. Steril. 85 (5), 1391–1397. 10.1016/j.fertnstert.2005.10.046 16600224

[B117] Wakai T. Zhang N. Vangheluwe P. Fissore R. A. (2013). Regulation of Endoplasmic Reticulum Ca(2+) Oscillations in Mammalian Eggs. J. Cell. Sci. 126 (Pt 24), 5714–5724. 10.1242/jcs.136549 24101727 PMC3860313

[B118] Wallace M. Cottell E. Gibney M. J. McAuliffe F. M. Wingfield M. Brennan L. (2012). An Investigation into the Relationship between the Metabolic Profile of Follicular Fluid, Oocyte Developmental Potential, and Implantation Outcome. Fertil. Steril. 97 (5), 1078–10848. 10.1016/j.fertnstert.2012.01.122 22365382

[B119] Williamson R. Lee D. Hagaman J. Maeda N. (1992). Marked Reduction of High Density Lipoprotein Cholesterol in Mice Genetically Modified to Lack Apolipoprotein A-I. Proc. Natl. Acad. Sci. U.S.A. 89 (15), 7134–7138. 10.1073/pnas.89.15.7134 1496008 PMC49660

[B120] Wree A. Eguchi A. McGeough M. D. Pena C. A. Johnson C. D. Canbay A. (2014). NLRP3 Inflammasome Activation Results in Hepatocyte Pyroptosis, Liver Inflammation, and Fibrosis in Mice. Hepatology 59 (3), 898–910. 10.1002/hep.26592 23813842 PMC4008151

[B121] Yao P. M. Tabas I. (2000). Free Cholesterol Loading of Macrophages Induces Apoptosis Involving the Fas Pathway. J. Biol. Chem. 275 (31), 23807–23813. 10.1074/jbc.m002087200 10791964

[B122] Yao P. M. Tabas I. (2001). Free Cholesterol Loading of Macrophages Is Associated with Widespread Mitochondrial Dysfunction and Activation of the Mitochondrial Apoptosis Pathway. J. Biol. Chem. 276 (45), 42468–42476. 10.1074/jbc.m101419200 11533046

[B123] Yates M. Kolmakova A. Zhao Y. Rodriguez A. (2011). Clinical Impact of Scavenger Receptor Class B Type I Gene Polymorphisms on Human Female Fertility. Hum. Reprod. 26 (7), 1910–1916. 10.1093/humrep/der124 21531995

[B124] Yesilaltay A. Dokshin G. A. Busso D. Wang L. Galiani D. Chavarria T. (2014). Excess Cholesterol Induces Mouse Egg Activation and May Cause Female Infertility. Proc. Natl. Acad. Sci. U. S. A. 111 (46), E4972–E4980. 10.1073/pnas.1418954111 25368174 PMC4246315

[B125] Yesilaltay A. Morales M. G. Amigo L. Zanlungo S. Rigotti A. Karackattu S. L. (2006). Effects of Hepatic Expression of the High-Density Lipoprotein Receptor SR-BI on Lipoprotein Metabolism and Female Fertility. Endocrinology 147 (4), 1577–1588. 10.1210/en.2005-1286 16410302

[B126] You J. S. Lim H. Seo J. Y. Kang K. R. Kim D. K. Oh J. S. (2021). 25-Hydroxycholesterol-Induced Oxiapoptophagy in L929 Mouse Fibroblast Cell Line. Molecules 27 (1). 10.3390/molecules27010199 PMC8746689 35011433

